# *Portulaca* Extract Attenuates Development of Dextran Sulfate Sodium Induced Colitis in Mice through Activation of PPAR*γ*

**DOI:** 10.1155/2018/6079101

**Published:** 2018-02-01

**Authors:** Rui Kong, Hui Luo, Nan Wang, Jingjing Li, Shizan Xu, Kan Chen, Jiao Feng, Liwei Wu, Sainan Li, Tong Liu, Xiya Lu, Yujing Xia, Yanhong Shi, Yingqun Zhou, Weigang He, Qi Dai, Yuejuan Zheng, Jie Lu

**Affiliations:** ^1^Department of Gastroenterology, Shanghai Tenth People's Hospital, Tongji University School of Medicine, Shanghai 200072, China; ^2^The School of Medicine, Soochow University, Suzhou 215006, China; ^3^Department of Gastroenterology, Shanghai Tenth Hospital, School of Clinical Medicine, Nanjing Medical University, Shanghai 200072, China; ^4^Department of Immunology and Microbiology, Shanghai University of Traditional Chinese Medicine, Shanghai 201203, China; ^5^The Eye Hospital, Wenzhou Medical University, Wenzhou City, Zhejiang 325027, China

## Abstract

*Portulaca oleracea* L. is a traditional Chinese medicine, which has been used as adjuvant therapy for inflammatory bowel disease (IBD). However, the mechanism of its activity in IBD still remains unclear. Since previous studies have documented the anti-inflammatory effect of peroxisome proliferator activated receptors-*γ* (PPAR-*γ*),* Portulaca* regulation of PPAR-*γ* in inflammation was examined in current study. Ulcerative colitis (UC) was generated by 5% dextran sulfate sodium (DSS) in mice and four groups were established as normal control, DSS alone, DSS plus mesalamine, and DSS plus* Portulaca*. Severity of UC was evaluated by body weight, stool blood form, and length of colorectum. Inflammation was examined by determination of inflammatory cytokines (TNF-a, IL-6, and IL-1a).* Portulaca* extract was able to attenuate development of UC in DSS model similar to the treatment of mesalazine. Moreover,* Portulaca* extract inhibited proinflammatory cytokines release and reduced the level of DSS-induced NF-*κ*B phosphorylation. Furthermore,* Portulaca* extract restored PPAR-*γ* level, which was reduced by DSS. In addition,* Portulaca* extract protected DSS induced apoptosis in mice. In conclusion,* Portulaca* extract can alleviate colitis in mice through regulation of inflammatory reaction, apoptosis, and PPAR-*γ* level; therefore,* Portulaca* extract can be a potential candidate for the treatment of IBD.

## 1. Introduction

Inflammatory bowel disease (IBD) consists of Crohn's disease and ulcerative colitis and has been considered as a global health threat to children and adults [1]. The distribution of IBD varies in geographical regions with high incidence rate in North American and Northern Europe; however, the morbidity remains stable in these regions. In the developing regions in the world such as Asia, the incidence and morbidity are increased every year [2]. Enormous efforts have been made to find an effective therapy for IBD although current drugs including sulfasalazine, mesalamine, and corticosteroids taken alone or in combination contribute to decelerating disease progression [3]. However, with current treatments and medications, some patients suffer from side effects and complications of these drugs such as weakened immunity, infectious diseases, or increased malignant potentiality. Therefore, a promising medication with no or mild side effects is needed for IBD treatment.

*Portulaca oleracea* L. (*Portulaca*) is a traditional Chinese herb praised with rich multiminerals, proteins, *α*-amyrin, *β*-carotene, terpenoids, vitamins, and fatty acids [4]. The medicinal properties and usage of* Portulaca* have been recorded in many ancient Chinese books [5]. Moreover, current researches reveal that* Portulaca* has several biological functions such as antibacteria, neuroprotection, anti-inflammation, antiatherosclerosis, and antitumor [6–10]. Furthermore, decoction of* Portulaca* has been used as a remedy for inflammatory bowel disease to alleviate symptoms of bloody stools, diarrhea, and abdominal pain in clinic. Several clinical trials have been conducted on the safety and effectiveness of* Portulaca*, which showed no adverse effects of* Portulaca*. Reports from animal studies also indicate low toxicity of* Portulaca* with LD_50_ value of 1853 mg/kg and high therapeutic index [11].

Peroxisome proliferator-activated receptor-*γ* (PPAR-*γ*) is an important member of the nuclear receptor superfamily. The essential role of PPAR-*γ* in acute inflammation [12], adipogenesis [13], and several carcinomas has been identified [14–16]. Moreover, PPAR-*γ* activation was found in various types of immune cells, including primary peritoneal macrophages, dendritic cells, and T cells [17]. Several studies have shown that activation of PPAR-*γ* could downregulate some vital proinflammatory cytokines, such as IL-1*β*, IL-2, IL-6, and TNF-*α* [18] as well as inhibition of NF-*κ*B phosphorylation in monocyte and other types of cells. Therefore, activation of PPAR-*γ* exerts a direct anti-inflammatory effect in IBD, while the mechanism of PPAR-*γ* activation remains to be further explored [19].

In the current study, the anti-inflammatory activity and mechanism of* Portulaca* in dextran sulfate sodium (DSS) induced UC animal model were explored.

## 2. Materials and Methods

### 2.1. Plant Material and Preparation of the Extracts

The* Portulaca* material was obtained from the Department of Traditional Chinese Medicine Pharmacy of the Shanghai Tenth People's Hospital. The herb was triturated into powder and water-soluble substance was extracted as described previously [20]. Briefly, the prepared powder was boiled in distilled water (80°C, 25 g/120 ml water, w/v) for 60 min and then smashed via ultrasonic concussion (3 cycles of 15 sec). The mixture of water and concussion was filtered using 2 mm pores strainer and the resultant material was lyophilized under vacuum.

### 2.2. Animals and Acute Colitis Induction

Forty adult female mice weighting 18–22 g were purchased from the Shanghai Laboratory Animal Co., Ltd. (Shanghai, China), and maintained in an environment of 25°C with controlled 12 h light/dark cycle. Mice were permitted free access to water and standard mouse chow. The animal protocol was approved by the Ethics Committee of the Tongji University, which meets the recommendations in the Guide for Care and Use of Laboratory Animals of the National Institutes of Health. The dextran sulfate sodium reagent (molecular weight: 36,000–50,000 from MP Biochemicals Solon, OH, USA) was diluted with drinking water to make final concentration of 3%.

The mice were divided into 4 groups at random:

1. Normal control: given normal food and water for 7 days
2. DSS group: 3% DSS + saline by gavage for 7 days
3. Mesalamine positive control group: 3% DSS + 7.4 mg/kg mesalamine daily by gavage for 7 days
4. *Portulaca* group: 3% DSS + 100 mg/kg daily by gavage for 7 days

During the period of modeling, mice weight, stool form, and stool occult blood were recorded every day to assess the disease activity index (DAI) of acute colitis; the evaluative criteria were clarified in Table 1.

### 2.3. Serum Cytokine Analysis

The serum levels of TNF-*α*, IL-1*β*, and IL-6 concentrations were determined by using ELISA Kits (Elabscience, Wuhan, China), respectively, according to the manufacturer's instruction. The OD values of absorbance at 450 nm were examined by ThermoMax microplate reader.

### 2.4. Determination of MPO (Myeloperoxidase) Activity in Serum Sample

Myeloperoxidase is found in activated neutrophils and macrophages, which plays a defensive role during inflammatory response via catalyzing hydrogen peroxide into hypochlorous acid. The serum samples reacted with the mixed solution including 3,3′-dimethoxybiphenyl-4,4′-diamine and hydrogen peroxide, pH 6.0, according to the MPO assay kit (Nanjing Jiancheng Bioengineering Institute, A044, China). The absorbance of control well and testing well at 460 nm was determined by microplate reader. The activity of MPO was calculated as the following formula:

(1) MPO  performance  U/L=testing  OD  values−control  OD  values11.3×sample  volume  L.

### 2.5. Fecal Occult Blood Testing

The form of stool was recorded and the severity of stool occult blood was analyzed by urine fecal occult blood test kit (Nanjing Jiancheng Bioengineering Institute, C027, China). A small amount of feces samples was picked and smeared on the slides. Add orthotolidine and hydrogen peroxide reagent on the surface of stool samples. The results were analyzed according to the indications: negative (−): the samples do not show color within 3 minutes; weakly positive (+): the samples show blue within 30 to 60 seconds; positive (++): the samples appear bluish green; strongly positive (+++): samples show mazarine immediately.

### 2.6. Gene Expression Analysis by Real-Time Reverse Transcriptase Polymerase Chain Reaction

Total RNA was extracted from fresh colorectum tissues by the Trizol reagent from the Thermo Fisher Scientific (Waltham, MA, USA) and washed by 75% ethanol. One microgram total RNA was transcribed into cDNA using reverse transcription kit (Takara Biotechnology, Dalian, China). The first transcribed cDNA was diluted ten times and applied to real-time polymerase chain reaction (SYBR Premix EX Taq, Takara Biotechnology) with the following cycle of denaturing at 94°C for 30 seconds, annealing at different temperature according to the primers for 30 seconds and elongation at 72°C for 45 seconds using a 7900HT Fast Real-Time PCR System (Thermo Fisher Scientific). All primers used in experiments were listed in Table 2.

### 2.7. Hematoxylin and Eosin Staining

Fresh colorectum tissues were fixed in formalin and embedded in paraffin. The tissue blot was cut into 4 *μ*m thick section for histological examination using hematoxylin and eosin (H&E). The injury index of histology was evaluated according to three aspects: the destructive degree of the intestine epithelium, the presence of the immune cell infiltration in intestine mucosa, and the infiltration degree of immune cell in submucosa. The histology score was depicted in Table 3. Two experienced pathologists made the assessment independently.

### 2.8. Western Blotting Analysis

Total protein was extracted from fresh colorectum tissue by cell lysis buffer with protease and phosphatase inhibitor mixture (New Cell & Molecular Biotech Co., Ltd., China). The protein quantification was determined by BCA methods (Beyotime, Shanghai, China). Equal amount of protein (25 ug) from each sample was loaded on to 12% SDS-PAGE. After separation through electrophoresis, the proteins were transferred onto PVDF membranes (Millipore Corp, Billerica, MA, USA). The membranes were then incubated with 5% nonfat milk in tris-buffered saline (TBS) to block nonspecific binding with antibody. The membranes were then incubated with primary antibodies at 4°C overnight. The primary antibodies used in experiments were as follows: *β*-actin, PPAR-*γ*, NF-*κ*B, pNF-*κ*B (all from Cell Signaling Technology, Danvers, MA, USA), Bcl-2, Bax (all from Proteintech Group, Inc., Chicago, IL, USA), and caspase 3 (Cell Signaling Technology, Danvers, MA, USA) with dilution of 1 : 1000, 1 : 1000, 1 : 1000, 1 : 500, 1 : 500, and 1 : 1000, respectively. After incubation with primary antibody, the membranes were washed three times with TBST and then incubated with the appropriate horseradish peroxidase-conjugated secondary antibodies. The signals were detected and the intensity of bands was analyzed by Odyssey.

### 2.9. Immunohistochemical Staining

Tissue specimen collected from mice was fixed in 4% paraformaldehyde overnight and embedded in paraffin. Sections (4 *μ*m) were dewaxed and hydrated through serial graded xylene ethanol. After being washed with phosphate-buffered saline (PBS) solution three times, antigen retrieval was performed using a microwave with heating to 95°C and cooling to room temperature (four times). Primary antibody PPAR-*γ* (Cell Signaling Technology, Danvers, MA, USA) was diluted into working concentration and then incubated with the slices at 4°C overnight. Following incubation of secondary antibody, DAB substrate was used as the chromogen. Positive expression areas were observed under microscope.

### 2.10. Statistical Analysis

The statistical analysis was performed using the SPSS 20.0 software package (IBM Corporation, Armonk, NY, USA). All values were expressed with the mean ± standard deviation using the Student–Newman–Keuls test or one-way analysis of variance. A *p* value < 0.05 was considered statistically significant. The positively stained areas of IHC were evaluated and analyzed using Image-Pro Plus 6.0 software (Media Cybernetics, Silver Spring, MD, USA).

## 3. Results

### 3.1. *Portulaca* Extract Decreases the Disease Activity Index, the Length of Colorectum, and Myeloperoxidase Activity of Dextran Sulfate Sodium Induced Colitis

**Figure 1:**
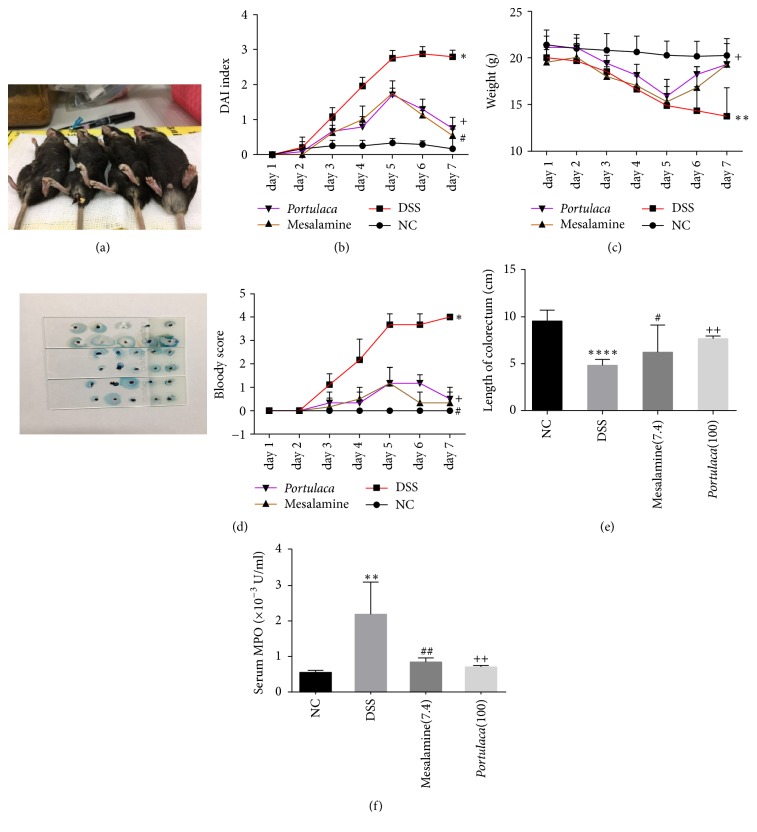
Effect of* Portulaca* treatment on disease severity of DSS-induced UC model. Panel (a) shows the appearance of representative mice from four experiment groups from left to right: control group, DSS-induced group, mesalamine group, and* Portulaca* group. Panel (b) displays the graph of disease activity index (DAI) of four groups at different days of the experiment. Panel (c) represents the weight of mice daily in different group. Panel (d) shows the image of fecal occult blood testing at 8th day with the darker color and more severe bloody stool and shows the score of bloody stool at different days of each group. Panel (e) represents the length of colorectum from four groups at 8th day after DSS administration. Panel (f) indicates the MPO activity in serum. (Data were presented as mean ± SD from six rats. *∗* indicates comparison between DSS and NC; # indicates comparison between mesalamine and DSS; + indicates the comparison between* Portulaca* and DSS. The number of symbols indicates significance of difference; for example, four symbols indicate *p* < 0.0001; three symbols indicate *p* < 0.001; two symbols indicate *p* < 0.01; and one symbol indicates *p* < 0.05.)

Since severity of colitis is evaluated by the DAI, MPO, and the length of colorectum, the effects of* Portulaca* extract on these parameters were examined. As shown in Figure 1(b), mice in DSS group have significant higher DAI than those in all other groups starting from day 3 (*p* < 0.05). Mice in* Portulaca* extract and mesalamine groups showed higher DAI starting from day 3 and reaching to the peak at day 5 compared to those in normal control. After day 5, DAI was gradually decreased although they were still higher than that in normal control. Since DAI consists of three aspects including weight loss, diarrhea, and the bloody stool scores. The weight loss and bloody stool scores were further evaluated and shown in Figures 1(c) and 1(d). DSS induced ulcerative colitis and significantly reduced body weight of the mice in DSS group (*p* < 0.01). However, the treatment of* Portulaca* extract and mesalamine significantly restored the body weights as shown in the* Portulaca* and mesalamine groups. In addition, there was a higher bloody stool score in DSS group than all other groups. Treatment of* Portulaca* and mesalamine reduced the score (Figure 1(d)).

The length of colorectum was an evaluation content of colitis, which means the total length from cecum to anus of mice. Compared with that of control group, there was a significant decrease in the length of colorectum in DSS group (*p* < 0.0001). But the length of colorectum in mesalamine and* Portulaca* group was longer than that in DSS group (Figure 1(e)). Moreover, myeloperoxidase (MPO) serves as an independent predictor for oxidative stress and inflammatory reactions. The average MPO activity in mice of control group was 0.56 ± 0.05 × 10^−3^ U/ml, while DSS exposure increased the MPO activity to 2.19 ± 0.82 × 10^−3^ U/ml. The treatment of mesalamine and* Portulaca* group reduced the MPO activity to 0.84 ± 0.11 × 10^−3^ U/ml and 0.71 ± 0.04 × 10^−3^ U/ml respectively (Figure 1(f)).

### 3.2. Effect of* Portulaca* Extract on Proinflammatory Cytokines Expression and Colorectum Injury

**Figure 2:**
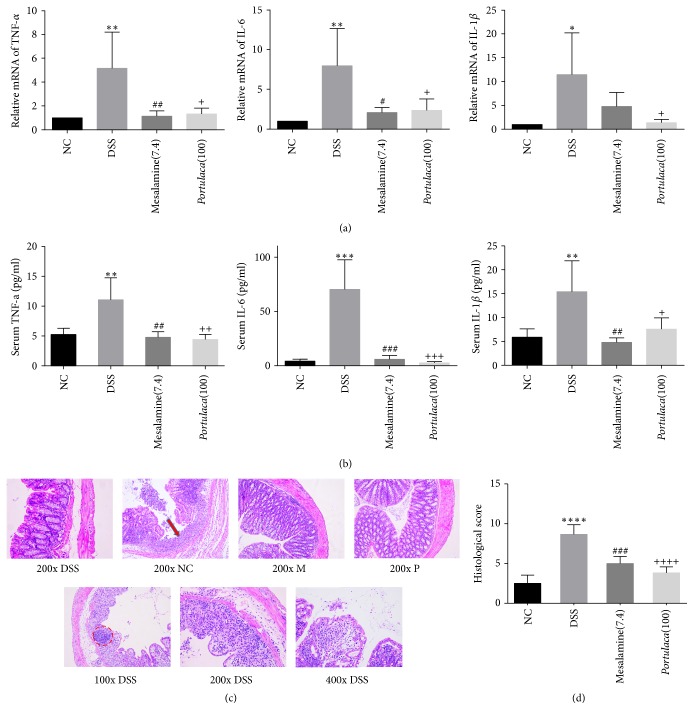
Effect of* Portulaca* extract on serum cytokines and tissue injury scores of DSS-induced acute colitis. Panel (a) indicates that the mRNA expressions of TNF-*α*, IL-6, and IL-1*β* were evaluated in each group with qRT- PCR. Panel (b) shows that the levels of TNF-*α*, IL-6, and IL-1*β* in serum were detected via ELISA assay. Panel (c) displays the H&E stained colon images (NC refers to negative control; DSS refers to DSS alone group; M refers to mesalamine group; P refers to* Portulaca* group). The dotted potion indicated the hyperplasia of lymphoid follicle; red arrow pointed the infiltration of inflammatory cells. Panel (d) indicates the histological scores of H&E stained colorectum images. (Data were presented as mean ± SD from six rats. *∗* indicates comparison between DSS and NC; # indicates comparison between mesalamine and DSS; + indicates the comparison between* Portulaca* and DSS. The number of symbols indicates significance of difference; for example, four symbols indicate *p* < 0.0001; three symbols indicate *p* < 0.001; two symbols indicate *p* < 0.01; and one symbol indicates *p* < 0.05.)

Since DSS induced ulcerative colitis, the expressions of three proinflammatory cytokines (TNF-*α*, IL-6, and IL-1*β*) were examined in the serum and colorectum tissue. As shown in Figures 2(a) and 2(b), there were significant increases in all three proinflammatory cytokines at both mRNA and protein levels (*p* < 0.05). Moreover, treatment of* Portulaca* and mesalamine significantly reduced the DSS-induced cytokine expression at both mRNA and protein levels (*p* < 0.05). Two independent experienced pathologists examined the tissue injury of colorectum. The injury score resulted from the evaluation of enterocyte lessons and inflammatory cell infiltration in mucosa and submucosa regions.  Figure 2(c) showed HE staining of colorectum tissues from mice of four different groups with magnification of 200x in the upper row and different magnification of colorectum in DSS mice. There was a significant increase in tissue injury score in DSS mice compared to all other mice (*p* < 0.0001). Treatment of* Portulaca* extract and mesalamine resulted in lesser enterocyte lesions and inflammatory cell infiltration in mucosa and submucosa (Figures 2(c) and 2(d)).

### 3.3. Regulation of NF-*κ*B and PPAR-*γ* by* Portulaca* Extracts in Colorectum

**Figure 3:**
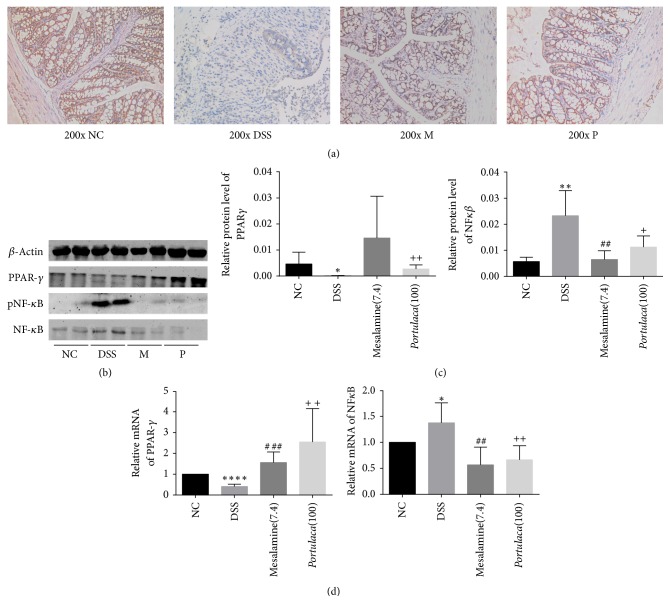
Effect of* Portulaca* extracts on NF-*κ*B and PPAR-*γ* expression in colonic tissue. Panel (a) represents the immunohistochemistry staining of PPAR-*γ*. (NC refers to negative control; DSS refers to DSS alone group; M refers to mesalamine group; P refers to* Portulaca* group, original magnification: ×200). Panel (b) shows the expression of PPAR-*γ*, NF-*κ*B, and pNF-*κ*B in colorectum tissues from western blot. Panel (c) shows the histograms of grey intensity for PPAR-*γ* and NF-*κ*B relative to *β*-actin. Panel (d) shows the relative mRNA expression of PPAR-*γ* and NF-*κ*B from quantitative real-time PCR. (Data are presented as mean ± SD from six experiments. *∗* indicates comparison between DSS and NC; # indicates comparison between mesalamine and DSS; + indicates the comparison between* Portulaca* and DSS. The number of symbols indicates significance of difference; for example, four symbols indicate *p* < 0.0001; three symbols indicate *p* < 0.001; two symbols indicate *p* < 0.01; and one symbol indicates *p* < 0.05.)

Both NF-*κ*B and PPAR-*γ* play an important role in inflammation, activation of NF-*κ*B is the critical step to increase the expression of different inflammatory cytokines such as TNF-*α*. Moreover, previous studies documented that PPAR-*γ* could antagonize inflammatory responses through inhibition of transcriptional activation of inflammatory response genes such as transcriptional factor NF-*κ*B. Therefore, the expressions of NF-*κ*B and PPAR-*γ* were examined in the study.  Figure 3(a) showed the staining of PPAR-*γ* in colorectum tissues from mice of four different groups. Significant reduction of PPAR-*γ* staining was observed in the colorectum of mice in DSS groups, while treatment of either* Portulaca* extract or mesalamine restored back the staining of PPAR-*γ* in the colorectum.  Figure 3(b) displayed both NF-kBp65 and PPAR-*γ* proteins by western blot and Figure 3(c) showed the mRNA levels of both NF-kB and PPAR-*γ*. There was significant decrease in PPAR-*γ* expression at both mRNA and protein levels in DSS group compared to that in normal control group (*p* < 0.05). Both* Portulaca* extract and mesalamine restored PPAR-*γ* levels back to that in normal control group. DSS treatment significantly induced NF-kB expression at both mRNA and protein levels (*p* < 0.05), while treatments of either* Portulaca* extract or mesalamine brought NF-kB levels back to that in normal control group.

### 3.4. Regulation of Apoptosis Protein in Colorectum by* Portulaca* Extract

**Figure 4:**
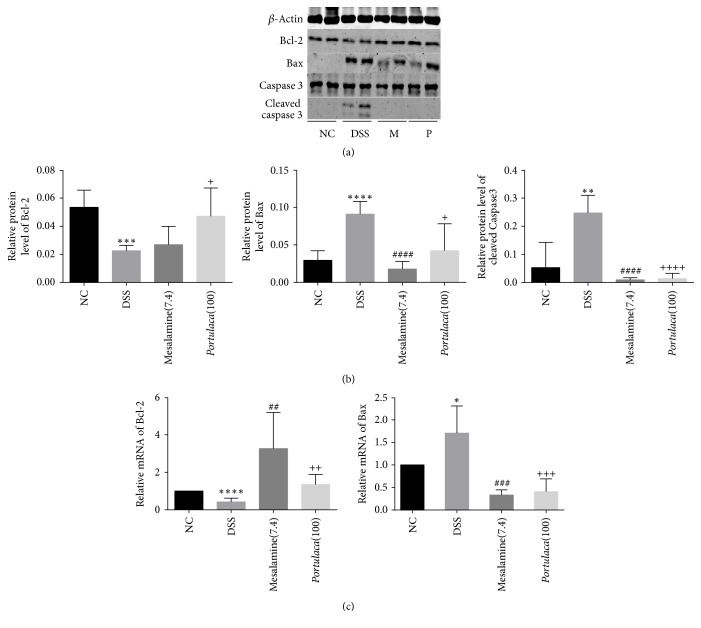
Effects of* Portulaca* extract on apoptosis in mice on inflammatory bowel disease. Panel (a) shows the protein level of Bcl-2, Bax, caspase 3, and cleaved caspase 3 in each group. Panel (b) represents the histogram of density for Bcl-2, Bax, and cleaved caspase 3. Panel (c) represents the relative mRNA level of Bcl-2 and Bax from quantitative real-time PCR, respectively. (Data are presented as mean ± SD from six experiments. *∗* indicates comparison between DSS and NC; # indicates comparison between mesalamine and DSS; + indicates the comparison between* Portulaca* and DSS. The number of symbols indicates significance of difference; for example, four symbols indicate *p* < 0.0001; three symbols indicate *p* < 0.001; two symbols indicate *p* < 0.01; and one symbol indicates *p* < 0.05.)

Inflammatory reaction usually results in cell death; however whether it is through apoptosis or necrosis remains to be explored. In the current study, three apoptotic proteins were examined including proapoptotic proteins (Bax and cleaved caspase 3) and antiapoptotic protein (Bcl-2). As shown in Figure 4, abundance of Bcl-2, Bax, and caspase 3 was documented by western blot analyses. There was significant decrease in Bcl-2 level (*p* < 0.001) but significant increase in Bax level in colorectum of mice in DSS group compared to all other groups (*p* < 0.0001). Although whole caspase 3 showed no change in the colorectum of mice in DSS group, the cleaved form of caspase 3 was significantly increased (*p* < 0.01). All these changes were corrected after treatment of either* Portulaca* extract or mesalazine. Moreover, mRNA levels of Bcl-2 and Bax showed the same changes as their proteins in the four different groups of mice.

## 4. Discussion

Inflammatory bowel disease commonly presents with intestine and extraintestine manifestations [21]. The typical symptom of IBD clinically is the blood and mucus mixed with stool. Other symptoms may include fevers, weight loss, arthritis, mucocutaneous lesions, and extraintestine presentations, which are useful for diagnosis, evaluating patient's condition and drug selection [22]. In the current study, weight loss and feces changes accompanied by blood and mucus mixed with stool were recorded daily to estimate the DAI (disease activity index) of the colitis. Significant high index of DAI in DSS-induced UC mice in the current study indicates successful establishment of UC model in mice. Moreover, with this model, therapeutic activity of* Portulaca* extract was also demonstrated showing that mice with treatment of* Portulaca* extract had significant lower DAI index than that without* Portulaca* treatment. Furthermore, with this successful model of UC, the effect of* Portulaca* extract on the length of colorectum was also documented. Mice with DSS-induced UC showed shorter colorectum than normal mice, which is consistent with the report of UC [23]. Treatment of* Portulaca* extract could also increase the length of colorectum in rats with DSS-induced colitis. The findings indicate that* Portulaca* extract could ameliorate general symptoms of IBD.

Currently, myeloperoxidase activity is considered as an independent indicator of inflammation and oxidative stress. This enzyme in leukocyte usually has the capacity to catalyze the hydrogen peroxide [24]. Clinically, myeloperoxidase activity has been used to predict inflammation and oxidative stress in renal failure [25], myocardial infarction [26], inflammatory vascular disease [27], and so on. Consistent with these reports, higher level of MPO was observed in mice of DSS treatment, which induces inflammation and ulcerative colitis in the intestine [28]. This elevation of MPO in serum is also consistent with intestine injury score observed in the colorectum tissues especially the infiltration of inflammatory cells in the mucosa and submucosa. These findings suggest that MPO could be an indicator for either DSS-induced colitis or clinical presentation of IBD.

Several immune cells and immune-regulatory proteins participated in the disturbance of intestine immune system, which account for activation and augmentation of inflammation cascade in IBD. The intestinal epithelial cells usually act as the first barrier for any intestinal disorders [29]. Once the epithelial cells are injured by external stress, intestine pathogens may enter the intestine, which triggers antigen-presenting cells and transform naive T cells into effector T cells such as Th1, Th17, Th2, and natural killer T cells. These cells are responsible for generating multiple types of proinflammatory cytokines [30]. Moreover, these cells could secret interferon *γ*, interleukin 1*β*, and other cytokines to activate macrophages, which in turn could secrete large amounts of cytokines including tumor necrosis factor *α*, interleukin 1, and interleukin 6. Furthermore, several cell types in the process such as dendritic cells, mast cells, and leucocytes can also promote the dysfunction of innate and adaptive immune response of inflammatory disease [31]. In current study, we found application of* Portulaca* could significantly reduce the expression levels of these cytokines including TNF-*α*, IL-6, and IL-1*β*. These findings suggested that* Portulaca* may affect T cell activation and participate in inflammation to some extent.

In present study, three proinflammatory cytokines (TNF-*α*, IL-6, and IL-1*β*) were examined. These cytokines are mainly released by macrophages and differentiated T cells [32]. In addition, TNF-*α* could also exert its inflammation response through upregulation of IL-1*β* and IL-6 and induce tissue damage through necrosis and apoptosis [33] and activate NF-*κ*B [34]. Moreover, all three cytokines are able to exert other functions by interacting with other molecules through different pathway. The findings of TNF-*α*, IL-1*β*, and IL-6 in the current study are in line with the reports in patients with either UC or CD [35, 36].

Except cytokines, transcriptional factors are also important in regulation of inflammation. The transcriptional factor NF-*κ*B is known to regulate the level of TNF-*α*, IL-6, and IL-1*β* during the progression of inflammatory bowel disease [37]. Moreover, NF-*κ*B can facilitate cell apoptosis through the upregulation of c-FLIP level while lowering the expression of antiapoptotic proteins such as Bcl-2, Bcl-xl, c-IAP1/2, and x-IAP [38]. In the present study, overexpressed TNF-*α*, IL-6, IL-1*β*, and pNF-*κ*B were observed in DSS treated mice, which indicate an enhanced inflammatory process and aggravated tissue damage. On the contrary, the addition of* Portulaca* extract reduced inflammation response and tissue damage indicating a role of* Portulaca* extract in prevention and treatment of inflammatory bowel disease.

PPAR-*γ* is another transcription factor, which plays an important role in adipogenesis, glucose metabolism [39], and anti-inflammatory reaction. Several reports suggest that PPAR-*γ* agonists may have beneficial effects for the inflammatory diseases, including mastitis, IBD, and arthritis [40–42]. Jiang et al. observed that PPAR agonist (15d-PGJ_2_) could abrogate the production of proinflammatory cytokines TNF-*α* and interleukin-6 [43]. Moreover, the NF-*κ*B activation is inhibited after PPAR-*γ* agonist due to changes of conformation in NF-kB subunits. The findings from the current study also observed that PPAR-*γ* level was increased in DSS-induced group following* Portulaca* extract intervention together with suppressed cytokines and NF-*κ*B.

The mechanism of apoptosis in IBD remains controversial. Some studies observed an increased apoptosis in lamina propria (LP) and epithelium, which contributed to the severity of UC [44]. Moreover, activation of PPAR-*γ* could attenuate the cascade of apoptosis [45]. In the current study, antiapoptosis protein (Bcl-2) was decreased and proapoptosis proteins (Bax and cleaved caspase 3) were increased in UC mice. However, treatment of* Portulaca* extract reverses apoptosis in the colorectum. This finding suggests further investigation of apoptosis and* Portulaca* in inflammatory bowel disease.

## 5. Conclusion

With the successful model of inflammatory bowel disease,* Portulaca* extract was demonstrated to reduce severity of the disease. The mechanism of* Portulaca* extract in alleviation of inflammatory bowel disease could be related to its regulation of inflammatory cytokines (TNF-*α*, IL-6, and IL-1*β*), transcription factors (NF-kB and PPAR-*γ*), and apoptosis. Therefore,* Portulaca* extract might be a potential agent for the treatment of patients with inflammatory bowel disease.

## Figures and Tables

**Table 1:** DAI assessment (disease activity index).

Weight loss %	Shape of stool	FOBT^*∗*^/ blood stool	Score
0	Normal	Normal	0
1–5	Shapeless stool	FOBT positive	1
6–10			2
11–15	Loose stool	Gross blood stool	3
>16			4

^*∗*^FOBT: fecal occult blood test.

**Table 2:** The sequence of primers used in experiments.

Gene	Primer sequence (5′-3′)	Annealing temperature (°C)
GADPH		
F	GGACCTCATGGCCTACATGG	57.6
R	TAGGGCCTCTCTTGCTCAGT	
IL-6		
F	GGCGGATCGGATGTTGTGAT	57.3
R	GGACCCCAGACAATCGGTTG	
IL-1*β*		
F	CTTTGAAGTTGACGGACCC	53.1
R	TGAGTGATACTGCCTGCCTG	
TNF-*α*		
F	CACCACCATCAAGGACTCAA	54.5
R	AGGCAACCTGACCACTCTCC	
PPAR-*γ*		
F	CCCAATGGTTGCTGATTACA	54.4
R	GGACGCAGGCTCTACTTTGA	
NF*κ*Bp65		
F	TGCGATTCCGCTATAAATGCG	60.2
R	ACAAGTTCATGTGGATGAGGC	
Bax		
F	AGACAGGGGCCTTTTTGCTAC	57.3
R	AATTCGCCGGAGACACTCG	
Bcl-2		
F	GAGCCTGTGAGAGACGTGG	57.3
R	CGAGTCTGTGTATAGCAATCCCA	

**Table 3:** Histology scoring.

Lesions of colonic epithelium	Granulocyte infiltration of intestine mucosa	Granulocyte infiltration of submucosa	Score
Normal	Normal	Normal	0
Cellular proliferation/anomalous crypt/absence of goblet cell	Mild	Mild to moderate	1
Absence of intestinal crypt (10%–50%)	Moderate	Severe	2
Absence of intestinal crypt (50%–90%)	Severe		3
Entire absence of intestinal crypt with integral epithelia			4
Mild to moderate ulceration epithelia			5
Severe ulceration			6
